# Ruptured coronary aneurysm with fatal bleeding: a tricky differential diagnosis

**DOI:** 10.1007/s10554-025-03402-0

**Published:** 2025-04-22

**Authors:** Pietro G. Lacaita, Gudrun M. Feuchtner

**Affiliations:** Department of Radiology, Innsbruck Medical University, Anichstrasse 35, Innsbruck, 6020 Austria

## Abstract

A 50 year-old-male presented with NSTEMI, a coronary fistula and a ruptured coronary aneurysm, which lead to fatal bleeding. The case highlights the pivotal role of coronary computed tomography angiography (CTA) in establishing the correct diagnosis based on CTA imaging findings.

**Supplementary Information:**

The online version contains supplementary material available at 10.1007/s10554-025-03402-0.

## Case description

A 50 year-old-male presented with acute chest pain and was diagnosed with NSTEMI, with a mild elevation in troponin T (Hs-Troponin 55.5 ng/dL, normal CK). Invasive coronary angiography (ICA) revealed a large coronary fistula and multiple aneurysms involving the LAD and CX. Coronary computed tomography angiography (CTA) identified a pulmonary artery fistula with multiple origins from the LAD and RCA. The largest LAD aneurysm (4 cm) exhibited an inhomogeneous wall thrombus, raising suspicion of rupture due to direct attachment to the pericardium and the presence of pericardial effusion. The patient remained hemodynamically stable and hospitalized. However, he suffered a sudden cardiac arrest only 12 h after CTA with unsuccessful cardiopulmonary resuscitation. Post-mortem examination confirmed LAD aneurysm rupture with direct pericardial communication and pericardial bleeding (1 litre).

## Discussion

**Fig. 1:**
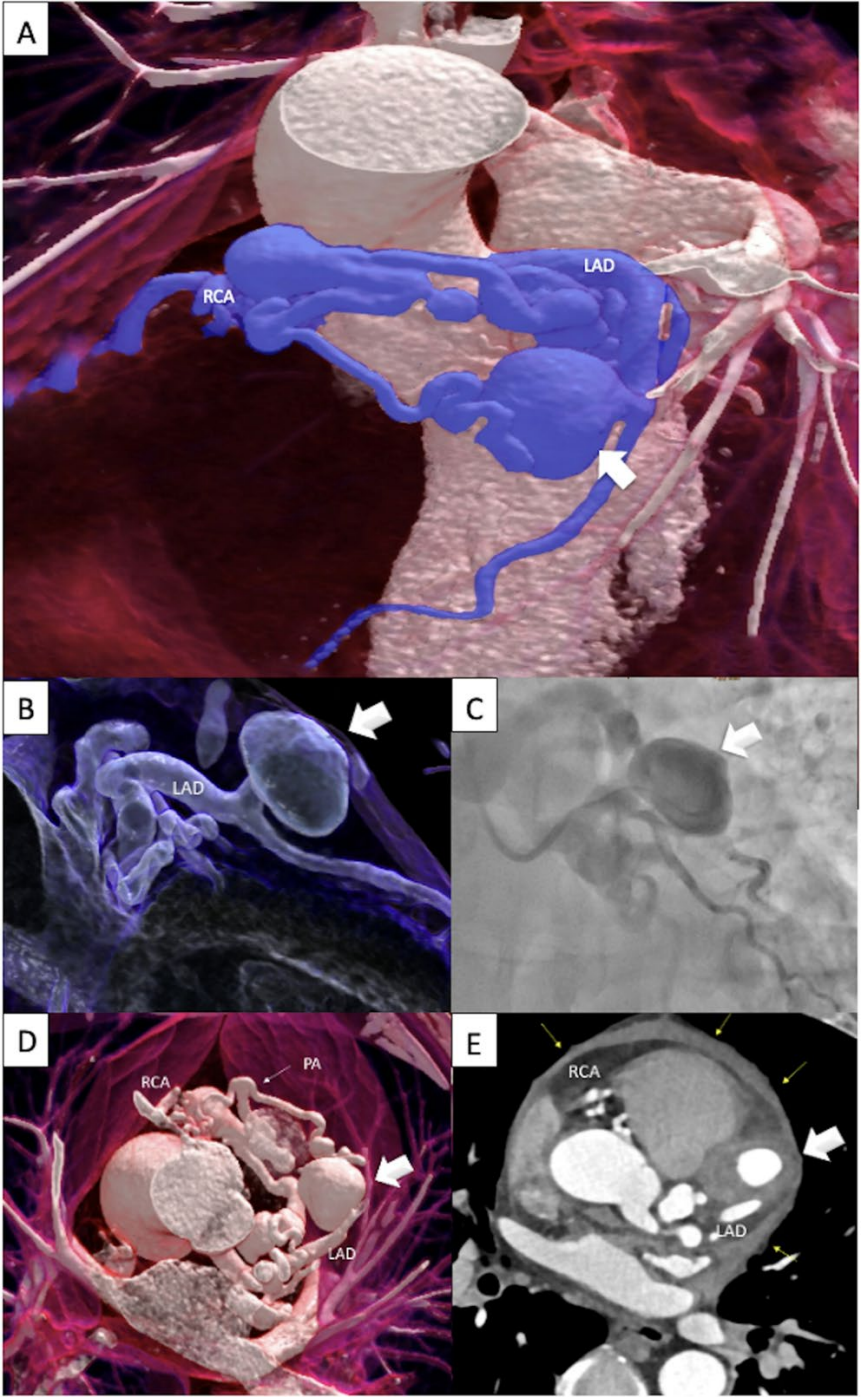
Ruptured Coronary Aneurysm (LAD). Panel A-B: Coronary CTA: 3D volume-rendered technique (VRT) image demonstrating the coronary fistula originating from the LAD with an associated large 4 cm size aneurysm (white arrow), connecting to the pulmonary artery (PA) and RCA. Panel C: Invasive coronary angiography depicting a large LAD aneurysm without evidence of active bleeding. Panel D. Coronary CTA 3DVRT: white arrow pointing at the LAD aneurysm. Panel E. Axial multiplanar reconstruction (MPR) showing an inhomogeneous wall thrombus within the LAD aneurysm, with CT attenuation values up to 69 HU, with direct attachment to the pericardium. The pericardial effusion (yellow arrows) demonstrated higher attenuation (60 HU) in the basal regions, while CT densities of the effusion in the mid-section and more cranially were consistency lower (30 HU). Therefore, hemorrhage into the pericardial space was supected (“sealed perforation”). The coronary artery fistula (CAF) had a large entry ostium into the pulmonary trunk, measuring 4.5 mm (white thin arrow). PA = pulmonary artery; RCA = right coronary artery; LAD = left anterior descending artery

Our case highlights the pivotal role of CTA in diagnosing complex coronary anomalies beyond what is visible on ICA, particularly in patients presenting with acute chest pain, to prevent fatal outcomes. Although ICA did not reveal active bleeding, CTA findings—a coronary artery fistula causing an LAD aneurysm with an inhomogeneous mural thrombus, pericardial attachment, and possible hemorrhagic effusion suggesting a sealed rupture—were the key for correct diagnosis(Fig. 1).

## Electronic supplementary material

Below is the link to the electronic supplementary material.

Supplementary Material 1

Supplementary Material 2

## Data Availability

No datasets were generated or analysed during the current study.

## Abbreviations

NSTEMI: Non-ST-Elevation Myocardial Infarction
Hs-Troponin: High-sensitivity Troponin
CK: Creatine Kinase
ICA: Invasive Coronary Angiography
LAD: Left Anterior Descending Coronary Artery
CX: Circumflex Coronary Artery
CTA: Computed Tomography Angiography
RCA: Right Coronary Artery
CX: circumflex artery
HU: Hounsfield Units
CAF: Coronary Artery Fistula
PA: Pulmonary Artery
MPR: Multiplanar Reconstruction
3DVRT / VRT: 3D Volume-Rendered Technique

